# Sex Chromosome Turnover in Moths of the Diverse Superfamily Gelechioidea

**DOI:** 10.1093/gbe/evz075

**Published:** 2019-04-08

**Authors:** Leonela Z Carabajal Paladino, Irena Provazníková, Madeleine Berger, Chris Bass, Nayanie S Aratchige, Silvia N López, František Marec, Petr Nguyen

**Affiliations:** 1 Biology Centre of the Czech Academy of Sciences, Institute of Entomology, České Budějovice, Czech Republic; 2 The Pirbright Institute, Surrey, United Kingdom; 3 University of South Bohemia, Faculty of Science, České Budějovice, Czech Republic; 4 Rothamsted Research, Department of Biointeractions and Crop Protection, Herts, United Kingdom; 5 University of Exeter, College of Life and Environmental Sciences, Biosciences, Penryn, Cornwall, United Kingdom; 6 Coconut Research Institute of Sri Lanka, Crop Protection Division, Bandirippuwa Estate, Lunuwila, Sri Lanka; 7 Instituto Nacional de Tecnología Agropecuaria, Instituto de Microbiología y Zoología Agrícola, Hurlingham, Buenos Aires, Argentina

**Keywords:** *Coleophora*, *Depressaria*, *Hofmannophila*, *Opisina*, *Phthorimaea*, *Sitotroga*

## Abstract

Sex chromosomes play a central role in genetics of speciation and their turnover was suggested to promote divergence. In vertebrates, sex chromosome–autosome fusions resulting in neo-sex chromosomes occur frequently in male heterogametic taxa (XX/XY), but are rare in groups with female heterogamety (WZ/ZZ). We examined sex chromosomes of seven pests of the diverse lepidopteran superfamily Gelechioidea and confirmed the presence of neo-sex chromosomes in their karyotypes. Two synteny blocks, which correspond to autosomes 7 (LG7) and 27 (LG27) in the ancestral lepidopteran karyotype exemplified by the linkage map of *Biston betularia* (Geometridae), were identified as sex-linked in the tomato leafminer, *Tuta absoluta* (Gelechiidae). Testing for sex-linkage performed in other species revealed that while LG7 fused to sex chromosomes in a common ancestor of all Gelechioidea, the second fusion between the resulting neo-sex chromosome and the other autosome is confined to the tribe Gnoreschemini (Gelechiinae). Our data accentuate an emerging pattern of high incidence of neo-sex chromosomes in Lepidoptera, the largest clade with WZ/ZZ sex chromosome system, which suggest that the paucity of neo-sex chromosomes is not an intrinsic feature of female heterogamety. Furthermore, LG7 contains one of the major clusters of UDP-glucosyltransferases, which are involved in the detoxification of plant secondary metabolites. Sex chromosome evolution in Gelechioidea thus supports an earlier hypothesis postulating that lepidopteran sex chromosome–autosome fusions can be driven by selection for association of Z-linked preference or host-independent isolation genes with larval performance and thus can contribute to ecological specialization and speciation of moths.

## Introduction

Sex chromosomes represent intriguing portions of the genome which play an important role in many evolutionary processes including sexual and intragenomic conflict and speciation (Masly and Presgraves 2007; Mank et al. 2014). Indeed, the formation of postzygotic isolation can be characterized by two empirical rules, both involving sex chromosomes, inferred from analyses of hybrid fitness. The first of these known as the large-X effect refers to the disproportionately large effect of the X chromosome compared with autosomes in introgression analyses of hybrid incompatibilities (Masly and Presgraves 2007; Dufresnes et al. 2016). The second, Haldane’s rule, which has proved to be one of the most robust generalizations in evolutionary biology, states that when in the F_1_ offspring of two different animal races one sex is absent, rare, or sterile, that sex is the heterogametic sex (Haldane 1922; Delph and Demuth 2016).

It was shown that larger and more heteromorphic sex chromosomes were associated with faster evolution of postzygotic isolation (Turelli and Begun 1997; Lima 2014). Sex chromosome size can increase via sex chromosome–autosome fusions, which result in so-called neo-sex chromosomes. These have been suggested to promote divergence in fish (Kitano et al. 2009; Kitano and Peichel 2012), mammals (Graves 2016), and moths (Nguyen et al. 2013; Nguyen and Carabajal Paladino 2016), although little is known about their functional role in this process. Neo-sex chromosomes also provide insight into the evolution of animal sex chromosomes (Pala et al. 2012; Bachtrog 2013; Natri et al. 2013), which are much older than the sex chromosome systems examined in plants (Charlesworth 2015). To identify the evolutionary forces driving sex chromosome–autosome fusions, the occurrence of derived multiple sex chromosome systems was recently analyzed in vertebrates (Pokorná et al. 2014; Pennell et al. 2015). These analyses yielded a striking pattern of a higher incidence of fusions in male heterogametic (♀XX, ♂XY) than female heterogametic (♀WZ, ♂ZZ) taxa. Moreover, it was shown that Y–autosome fusions occur most frequently. Theoretical models suggested that a combination of two or more evolutionary forces, such as underdominance of the fusions, male-biased mutation rates for fusions, and female-biased reproductive sex ratio, is needed to explain the asymmetry between the Y and W chromosomes (Pennell et al. 2015; Kirkpatrick 2017).

Moths and butterflies (Lepidoptera), together with their sister order caddisflies (Trichoptera), constitute the most speciose lineage with female heterogamety. In their overview of 40 lepidopteran species with identified sex chromosomes, Traut et al. (2007) listed 12 moths with multiple sex chromosomes. Since then, more neo-sex chromosome systems have been reported in this order (Nguyen et al. 2013; Šíchová et al. 2013, 2015, 2016; Smith et al. 2016; Fraïsse et al. 2017; Mongue et al. 2017; Traut et al. 2017; Picq et al. 2018).

Some of the derived sex chromosome systems correspond to a conspicuously large sex chromosome pair (Nguyen et al. 2013; Šíchová et al. 2013; Mongue et al. 2017; Picq et al. 2018), which suggests that both W and Z sex chromosomes fused with an autosome. Similar large chromosome pairs were also observed in representatives of the families Pyralidae, Oecophoridae, and Gelechiidae with reduced chromosome numbers, but were considered autosomal fusion products (Ennis 1976). Carabajal Paladino et al. (2016), however, showed that the large chromosome pair corresponds to sex chromosomes in an invasive gelechiid pest, the tomato leafminer *Tuta absoluta* (Gelechiidae).

To test for the presence of neo-sex chromosomes in their genomes, we examined the karyotypes of several pests of the diverse superfamily Gelechioidea, which contains ∼18,500 species (van Nieukerken et al. 2011) and comprises among others the above-mentioned Oecophoridae and Gelechiidae families. Our results confirmed a sex chromosome–autosome fusion, which occurred in a common ancestor of all three main lineages of Gelechioidea, namely the Gelechiid, Scythridid, and Depressariid assemblages (Sohn et al. 2016). A synteny block involved in the fusion was identified as an autosome homoeologous to the chromosome 7 of the ancestral karyotype represented by the peppered moth *Biston betularia* (Geometridae) (cf. Van’t Hof 2013). Furthermore, we discovered another fusion between the neo-sex chromosomes and homoeologue of the *B. betularia* chromosome 27 within the tribe Gnorimoschemini (Gelechiinae). A potential role of the sex chromosome turnover in the divergence of Gelechioidea is discussed.

## Materials and Methods

### Insects

Representatives of five families within Gelechioidea were either obtained from laboratory stocks or collected from natural populations. A laboratory stock of the potato tuber moth, *Phthorimaea operculella* (Gelechiidae), was provided by the Atomic Energy Commission of Syria (Damascus, Syria). Larvae were reared on wax-coated potato slices as described in Saour and Makee (1997). Cultures of the Angoumois grain moth, *Sitotroga cerealella* (Gelechiidae), from the Instituto de Microbiología y Zoología Agrícola (IMYZA), Instituto Nacional de Tecnología Agropecuaria (INTA) (Buenos Aires, Argentina), and the Institute for Biological Control JKI, Federal Research Centre for Cultivated Plants (Darmstadt, Germany) were kept on wheat grains (Méndez et al. 2016). A laboratory colony of the tomato leafminer, *T.absoluta* (Gelechiidae), from IMYZA, INTA was maintained on potted tomato plants under the conditions detailed in Cagnotti et al. (2012). Specimens of the coconut black-headed caterpillar, *Opisina arenosella* (Xylorictidae), were obtained from the colony maintained on coconut leaflets at the Crop Protection Division of the Coconut Research Institute of Sri Lanka (Lunuwila, Sri Lanka). The larch case-bearer *Coleophora laricella* (Coleophoridae) and the brown house-moth *Hofmannophila pseudospretella* (Oecophoridae) were collected as larvae from wild populations in Levín (Lišov, Czech Republic). The dingy flat-body moth *Depressaria daucella* (Depressaridae) was collected as larvae and pupae in Slapy u Tábora (Tábor, Czech Republic). The material obtained in the field was immediately processed for its future analysis, and barcoded using a fragment of the *cytochrome c oxidase subunit I* (*COI*) gene as described in Hebert et al. (2004). The sequences obtained were checked in the BOLD animal identification database (Ratnasingham and Hebert 2007) to confirm the identity of the specimens (for accession numbers of the sequences, see supplementary table S1, Supplementary Material online).

### Processing of the Insects

Spread chromosome preparations were made from wing imaginal discs, testes, or ovaries of the last instar larvae of all species using the method of Traut (1976) with slight modifications detailed in Šíchová et al. (2013). For *D. daucella*, preparations were also made from ovaries of female pupae. The preparations were dehydrated in an ethanol series (70%, 80%, and 100%, 30 s each) and stored at −20 °C.

Nucleic acids were isolated from larvae or pupae. Given the size of the specimens, total RNA was recovered using the NucleoSpin RNA II (Macherey-Nagel, Düren, Germany) kit, RNA blue (Top-Bio, Prague, Czech Republic), or RNAzol (Sigma–Aldrich, St. Louis, MO). The first-strand cDNA was then synthesized by random or oligo-dT primed SuperScript III Reverse Transcriptase (Invitrogen, Carlsbad, CA). Genomic DNA (gDNA) was extracted either by the NucleoSpin Tissue kit (Macherey-Nagel) or the MagAttract HMW DNA Kit (Qiagen, Hilden, Germany) and if needed, amplified by illustra GenomiPhi HY DNA Amplification Kit (GE Healthcare, Milwaukee, WI).

### Fluorescence In Situ Hybridization Experiments

To identify sex chromosomes genomic in situ hybridization (GISH) was performed as described in Yoshido et al. (2005). Amplified male gDNA was fragmented by heating to 99 °C for 10 min in a TProfessional TRIO thermocycler (Biometra, Göttingen, Germany), and used as a species-specific competitor DNA (Šíchová et al. 2013). Female gDNA was labeled with ﬂuorescein-12-dUTP (Jena Bioscience, Jena, Germany) using the nick translation protocol of Kato et al. (2006) with 3.5-h incubation at 15 °C. To accurately determine chromosome numbers, fluorescence in situ hybridization (FISH) with (TTAGG)_*n*_ telomeric probes (tel-FISH) was performed either alone or in combination with GISH as described in Yoshido et al. (2005) and Šíchová et al. (2015). Unlabeled (TTAGG)_*n*_ telomeric probes were prepared by nontemplate PCR according to Sahara et al. (1999) and labeled with Cy3-dUTP (Jena Bioscience) using the same nick translation protocol as above, but with 1-h incubation at 15 °C. For each slide, the hybridization mixture contained unlabeled fragmented male gDNA (3 µg) and female fluorescein-labeled gDNA (500 ng), and/or Cy3-labeled telomeric probe (200 ng), and sonicated salmon sperm DNA (25 µg). The preparations were counterstained with 0.5 mg/ml DAPI (4’,6-diamidino-2-phenylindole; Sigma–Aldrich) in antifade based on DABCO (1,4-diazabicyclo[2.2.2]octane; Sigma–Aldrich) (for composition, see Traut et al. 1999).

Preparations from FISH experiments were observed in a Zeiss Axioplan 2 microscope (Carl Zeiss, Jena, Germany) equipped with appropriate fluorescence filter sets. Black-and-white images were captured with an Olympus CCD monochrome camera XM10 equipped with cellSens 1.9 digital imaging software (Olympus Europa Holding, Hamburg, Germany). The images were pseudocolored and superimposed with Adobe Photoshop CS6 (Adobe Systems, San Jose, CA).

### Screening for *T. absoluta* Sex-Linked Genes

The sex-linkage of selected genes was tested by means of quantitative PCR (qPCR) using male and female gDNA as template and autosomal gene as a reference (Nguyen et al. 2013; Dalíková et al. 2017). The selected genes were orthologous to markers for all the chromosomes of the ancestral karyotype represented by the *B.betularia* (Geometridae) linkage map (Van’t Hof 2013) and the *Melitaea cinxia* (Nymphalidae) genome (Ahola et al. 2014) (supplementary table S2, Supplementary Material online). Primers were designed using available *T. absoluta* transcriptome sequences (Berger et al. 2016). The 1:1 (female:male) ratio of the used autosomal reference genes, *elongation factor 1 alpha* (*EF-1a*) and *acetylcholinesterase 1* (*Ace-1*) (using *Ace-1* as target and *EF-1a* as reference), and the 1:2 ratio of the Z-linked control gene *kettin* (*ket*) (using *Ace-1* as reference) were verified before analyzing other markers. The genes were tested in triplicates of three independent samples of both male and female gDNAs. Amplification efficiencies (*E*) of primer pairs were determined from the slope of the standard curve generated by plotting the threshold cycle (Ct) values against the log-concentrations of serial dilutions of male and female gDNAs. The female-to-male (F:M) ratio for each gene was calculated for each female as [(1 + *E*_target_) ^ (Average_Ct_target_male_ − Ct_target_female_)] / [(1 + *E*_reference_) ^ (Average_Ct_reference_male_ − Ct_reference_female_)], and then compared with the expected values of 1 and 0.5 corresponding to autosomal position and sex-linkage, respectively, by means of one-sample *t*-test using R (R Core Team 2013) (supplementary table S3, Supplementary Material online). Composition of the reaction, cycling conditions, and sequences of forward and reverse primers are detailed in supplementary tables S3 and S4, Supplementary Material online.

Once these control genes were validated, the marker genes (supplementary table S2, Supplementary Material online) were analyzed using one biological replicate per sex with three technical replicates per gDNA sample. In this case, the F:M ratio was calculated using the delta delta Ct method as 2 ^ [(Ct_target_female_ − Ct_reference_female_) − (Ct_target_male_ − Ct_reference_male_)], which is a simplified version of the aforementioned formula that assumes *E *=* *1 for all genes. The obtained values were considered for analysis only if *ket* and/or the reference genes (*EF-1a* and *Ace-1*) compared with each other provided the expected and previously corroborated 0.5 and 1 values, respectively. The experiments were carried out at least three times, using both reference genes, *EF-1a* and *Ace-1*. All reactions were performed in a final volume of 25 µl using SYBR Premix Ex *Taq* II (Perfect Real Time) (TaKaRa, Otsu, Japan) and a final concentration of primers of 0.2 mM for both the target and reference genes (except for *pixie ATP-binding cassette subfamily E member 1* [*Pix*] when a final concentration of 0.3 mM was used for the primers of the target gene). The cycling conditions included an initial denaturation at 95 °C for 3 min, then 45 cycles of 94 °C for 30 s, 60 °C for 30 s, 72 °C for 30 s, a final denaturation of 95 °C for 15 s, and then an increase of temperature from 65 to 95 °C with increments of 0.5 °C for 5 s for the generation of melting curves. The sequences of forward and reverse primers are detailed in supplementary table S2, Supplementary Material online.

All qPCR experiments were performed in FrameStar 96 well plates (Institute of Applied Biotechnologies [IAB], Prague, Czech Republic) covered by µltraAmp Plate Sealers (Sorenson BioScience, Salt Lake City, UT) or qPCR adhesive foil (IAB) using a C1000 Thermal cycler CFX96 Real-Time System (Bio-Rad, Hercules, CA).

### Cloning of Genes of Interest in Other Gelechioid Species

The genes of interest included the reference genes *EF-1a* and *Ace-1*, together with the markers proven to be sex-linked in *T. absoluta*, namely *Pix* and *chitinase h* (*Chit*) for the chromosome homoeologous to *B. betularia* linkage group (BbLG) 7, and *90-kDa heat shock protein* (*Hsp90*) and *twitchin* (*Tw*) for the chromosome homoeologous to BbLG27 (see results for details). Degenerate primers (supplementary table S5, Supplementary Material online) were designed for regions of coding sequences conserved between Lepidoptera and other insect species, and used for RT-PCR amplification of partial sequences with the first-strand cDNA as a template. Amplified fragments were cloned using pGEM-T Easy Vector System (Promega, Madison, WI) or CloneJET PCR cloning kit (Thermo Fisher Scientific, Waltham, MA), and confirmed by Sanger sequencing. The obtained sequences were deposited in GenBank (for accession numbers, see supplementary table S1, Supplementary Material online) and used for the design of species-specific primers for qPCR experiments (supplementary table S4, Supplementary Material online).

### Quantitative Analysis of Gene Dose in the Other Gelechioid Species

Quantitative PCR experiments using male and female gDNAs as template were conducted in *S. cerealella*, *P. operculella*, *C. laricella*, *O. arenosella*, *H. pseudospretella*, and *D. daucella* to test for the sex-linkage of *Pix*, *Chit*, *Hsp90*, and *Tw*. Male and female gene doses of the target genes were compared with *EF-1a* and/or *Ace-1*. Three technical and three biological replicates were used per experiment. Composition of the reactions, cycling conditions, and sequences of forward and reverse species-specific primers are detailed in supplementary tables S3 and S4, Supplementary Material online. The F:M ratio was calculated including the *E* value of the primers, according to the formula mentioned earlier, and then compared with the expected values of 1 and 0.5 corresponding to autosomal position and sex-linkage, respectively, by means of one-sample *t*-test using R.

## Results

### Barcoding of Collected Specimens

The field collected larvae used for chromosome preparations were barcoded using a partial sequence of *COI*. The sequences confirmed the classification of *H.pseudospretella* (Oecophoridae) and *D.daucella* (Depressaridae) with 100% identity with their respective records in the BOLD database. In the case of the Coleophoridae specimens, our search retrieved matches with *C.laricella* and *Coleophorasibiricella*. Since the geographical distribution of both species does not overlap in the Czech Republic (Laštůvka and Liška 2011), we considered the samples as *C. laricella* in our analysis. The consensus sequences of the *COI* fragments for all species examined were deposited in the GenBank database under accession numbers detailed in supplementary table S1, Supplementary Material online.

### Karyotype Analyses

In comparison with the most common and ancestral lepidopteran chromosome number *n* = 31 (see Discussion for details), all species of Gelechioidea studied herein showed a reduced chromosome number ranging from *n* = 28 to *n* = 30. These values are in concordance with those observed in other representatives of the superfamily, which shows a modal chromosome number of *n* = 29 in 15 out of 33 studied species (supplementary table S6, Supplementary Material online). FISH with the telomeric probe marking chromosome ends was used to accurately count chromosome numbers in some of the examined species (cf. Šíchová et al. 2015; not shown).

In the Gelechiid assemblage, a complete karyotype analysis including the identification of sex chromosome constitution analysis has not been performed except for *P.operculella* (Gelechiidae) (Bedo 1984; Makee and Tafesh 2006; supplementary table S6, Supplementary Material online). In the present study, we analyzed two representatives of the family Gelechiidae, namely *T.absoluta* and *S.cerealella.*

**Fig. 1.**
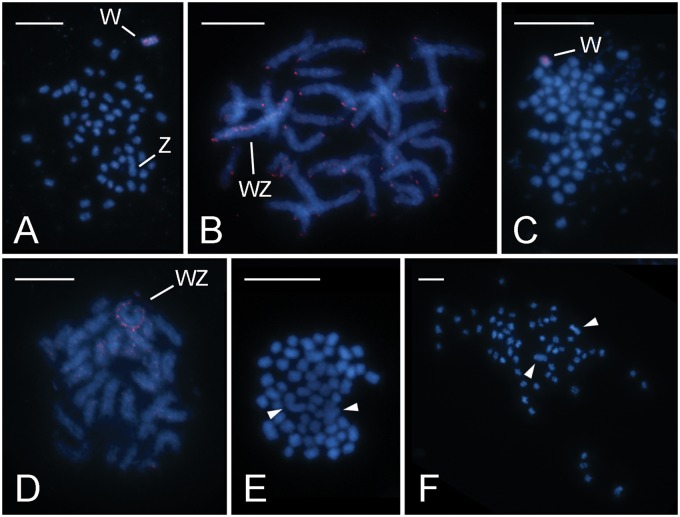
—Cytogenetic analysis of representatives of the Gelechiid and Scythridid assemblages. Chromosomes were counterstained with DAPI (blue); female derived genomic probes (*A–D*) were labeled by Cy3 (red). (*A* and *B*) GISH in *Tuta absoluta* (Gelechiidae, Gelechiid assembl.): (*A*) female mitotic metaphase consisting of 2*n* = 58 elements; note that the W chromosome is one of the two largest chromosomes in the complement; (*B*) female pachytene nucleus; the probe labeled the W chromosome in the WZ bivalent and chromosome ends of most bivalents. (*C* and *D*) GISH in *Sitotroga cerealella* (Gelechiidae, Gelechiid assembl.): (*C*) female mitotic metaphase consisting of 2*n* = 60 chromosomes; the W chromosome is not conspicuously larger than the other chromosomes; note DAPI-stained small rod-shaped bodies, probably corresponding to bacteria; (*D*) late pachytene female nucleus; the probe identified the W chromosome in the WZ bivalent. (*E* and *F*) Mitotic complements of *Coleophora laricella* (Coleophoridae, Scythridid assembl.) stained with DAPI: (*E*) male mitotic metaphase consisting of 2*n* = 58 chromosomes; note a pair of large chromosomes (arrowheads); (*F*) female mitotic metaphase comprising 2*n* = 58 chromosomes; note a pair of large chromosomes (arrowheads). Bar = 10 µm.

In *T. absoluta*, Carabajal Paladino et al. (2016) determined the haploid chromosome number of *n* = 29 and identified the largest elements as sex chromosomes morphometrically. In the present study, we identified the W chromosome by means of GISH, in which the labeled female gDNA-derived probe was hybridized to chromosomes in excess of unlabeled male competitor DNA. In mitotic complements, hybridization signals clearly highlighted one chromosome of the large pair. GISH thus confirmed that this is the W chromosome and implied that the other large element represents the Z chromosome (fig. 1*A*). The probe produced signals scattered along the W chromosome with notable exception of one subtelomeric and one interstitial gap in pachytene nuclei, and in some experiments also highlighted the chromosome ends (fig. 1*B*).

The haploid chromosome number of *n* = 30 was previously described for males of *S. cerealella* (Lukhtanov and Kuznetsova 1989). We confirmed the chromosome number in mitotic complements, 2*n* = 60, in males (not shown) as well as in females (fig. 1*C*). Furthermore, we used GISH to identify the female-specific W chromosome in mitotic complements (fig. 1*C*). In most mitotic metaphases, the W chromosome was not clearly discernible by size. In order to improve the resolution, GISH experiments were performed on female preparations of elongated pachytene bivalents. These experiments provided a more informative labeling pattern of the female genomic probe on the W chromosome. Hybridization signals of the probe were scattered along the entire W chromosome (fig. 1*D*). Interestingly, chromosome preparations obtained from the Argentinian *S. cerealella* females were contaminated with small DAPI-positive bodies, most likely corresponding to some bacteria present in the ovaries (fig. 1*C*).

*Coleophora laricella* (Coleophoridae) was the only representative of the Scythridid assemblage examined in this study. Mitotic metaphase complements consisted of *n* = 29 in both males and females of this species. The karyotype of both sexes comprised a conspicuously large chromosome pair (fig. 1*E* and *F*). Surprisingly, GISH provided weak or no hybridization signals in mitotic nuclei (not shown). GISH carried out on less condensed female pachytene chromosomes failed to identify a W-chromosome as well (not shown). Telomeric FISH combined with GISH was used as a control and yielded clear telomeric but no GISH signals (not shown). So it seems that our negative GISH results are not artifactual but rather point to an exceptional molecular composition of the *C. laricella* W chromosome. The W chromosome of *C. laricella* presumably does not differ from the rest of the genome in that it comprises a diverse spectrum of ubiquitous repeats present at low abundance.

**Fig. 2.**
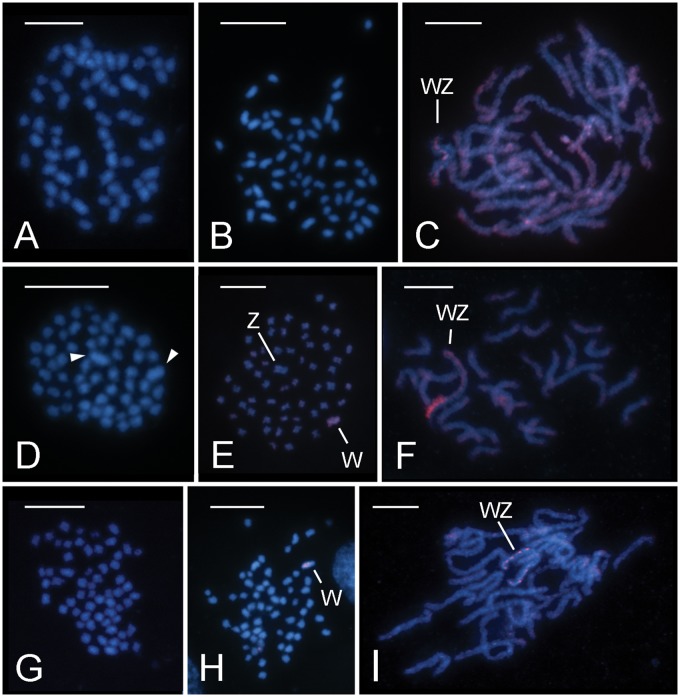
—Cytogenetic analysis of representative of the Depressariid assemblage. Chromosomes were counterstained with DAPI (blue); female-derived genomic probes (*C*, *E*, *F*, *H*, *I*) were labeled by Cy3 (red). (*A–C*) *Opisina arenosella* (Xyloryctidae): (*A*) male mitotic metaphase consisting of 2*n* = 60 elements; (*B*) female mitotic metaphase consisting of 2*n* = 60 chromosomes; (*C*) GISH on female pachytene nucleus; note the hybridization signals on all bivalents either along the entire chromosomes or with preference for subterminal regions; the WZ bivalent is identified by the signal intensity that differs between the W and Z chromosome threads, as well as by the characteristic pairing of the longer Z chromosome twisted around the much shorter W chromosome. (*D–F*) *Hofmannophila pseudospretella* (Oecophoridae): (*D*) male mitotic metaphase consisting of 2*n* = 56 chromosomes; note the two largest chromosomes (arrowheads); (*E* and *F*) GISH on female chromosome preparations; (*E*) female mitotic metaphase comprising 2*n* = 56 chromosomes; note that GISH identified the W chromosome as one of the two largest chromosomes; (*F*) female pachytene nucleus; note the size and bipartite organization of the WZ bivalent with about one-third of the W chromosome thread strongly labeled with the probe. (*G–I*) *Depressaria daucella* (Depressariidae): (*G*) male mitotic metaphase comprising 2*n* = 60 chromosomes; note that there is no conspicuously larger chromosome pair; (*G–I*) GISH on female chromosome preparations; (*H*) female mitotic metaphase consisting of 2*n* = 60 elements with the W chromosome identified by the probe; (*I*) female pachytene nucleus; note the WZ bivalent showing scattered hybridization signals of the probe on the W chromosome thread. Bar = 10 µm.

Within the Depressariid assemblage, three species, namely *O.arenosella* (Xyloryctidae), *H. pseudospretella* (Oecophoridae), and *D. daucella* (Depressaridae), were investigated. In *O. arenosella*, the diploid chromosome complement consisted of 2*n* = 60 chromosomes in both males (fig. 2*A*) and females (fig. 2*B*). No elements showed significant size differences in *O. arenosella*, with all chromosomes decreasing gradually in size, which is typical for lepidopteran karyotypes (fig. 2*A* and *B*). In addition, no mitotic chromosome was reliably discerned by GISH in this species as the female-derived genomic probe labeled all chromosomes more or less with the same intensity (not shown). In pachytene, the probe labeled all bivalents, some along the entire chromosome length and some preferentially in subterminal regions. However, one bivalent was conspicuous by its heteromorphic staining with one of its threads intensively stained while the other was not (fig. 2*C*). It is reasonable to assume that this bivalent corresponds to the WZ sex chromosome pair. The absence of hybridization signals on the Z chromosome is likely a result of its hemizygosity in females from which the GISH probe was derived. The sex chromosome bivalent identity was further supported by its meiotic pairing pattern, as the signal-free chromosome typically twisted several times around its labeled partner (fig. 2*C*). This was due to the size difference between the sex chromosomes with the W being much shorter than the Z chromosome (cf. Marec and Traut 1994).

In *H. pseudospretella*, a reduced diploid chromosome number of 2*n* = 56 with two large chromosomes was observed in mitotic metaphase nuclei of both sexes (fig. 2*D* and *E*). The female-derived genomic probe clearly highlighted one of the large chromosomes in female mitotic metaphase complements (fig. 2*E*). Thus, the largest chromosome pair most likely comprises the sex chromosomes. However, in female pachytene nuclei, a WZ bivalent could not be identified without the use of GISH. This method revealed a bipartite organization of the W chromosome, as it strongly labeled one terminal region corresponding to roughly one-third of the sex chromosome bivalent (fig. 2*F*).

The diploid chromosome number was 2*n* = 60 in both sexes of *D. daucella*. Neither male nor female mitotic complement comprised any notably larger chromosome (fig. 2*G* and *H*). GISH identified one of the larger chromosomes as the W chromosome in the *D. daucella* female mitotic metaphase complements (fig. 2*H*). In female pachytene nuclei, the WZ bivalent was easily discerned by the heterochromatic W thread (not shown). GISH showed scattered hybridization signals colocalizing with DAPI positive blocks on the W chromosome (fig. 2*I*).

### Identification of Sex-Linked Synteny Blocks in Gelechioidea

To identify sex-linked synteny blocks, the sex-linkage of *T. absoluta* genes was tested by qPCR using male and female gDNA as template. This method can detect hemizygosity of Z-linked markers caused either by the absence or molecular degradation of their W-linked gene copies (Nguyen et al. 2013; Dalíková et al. 2017). The variable female-to-male (F:M) ratio between the selected reference genes *EF-1a* and *Ace-1*, using *Ace-1* as target and *EF-1a* as reference, was 1.000 ± 0.102 (SE), which statistically differed from 0.5 (*P *<* *0.05) but not from 1 (*P *>* *0.05) (supplementary table S3, Supplementary Material online). The F:M ratio for *ket*, using *Ace-1* as reference, gave a value of 0.498 ± 0.090, which significantly differed from 1 (*P *<* *0.05) but not from 0.5 (*P *>* *0.05) (supplementary table S3, Supplementary Material online). These results indicated that females and males had the same copy number of both *Ace-1* and *EF-1a* genes, and that females had half the number of copies of *ket* with respect to males, which was expected as this gene represents a standard marker for the lepidopteran Z chromosome (cf. Nguyen et al. 2013; Van’t Hof 2013). The analysis thus confirmed that the *Ace-1* and *EF-1a* genes are autosomal and can be used as reference genes for further studies. It also proved *ket* as a good control gene for the screening of sex-linked markers in *T. absoluta*.

**Fig. 3.**
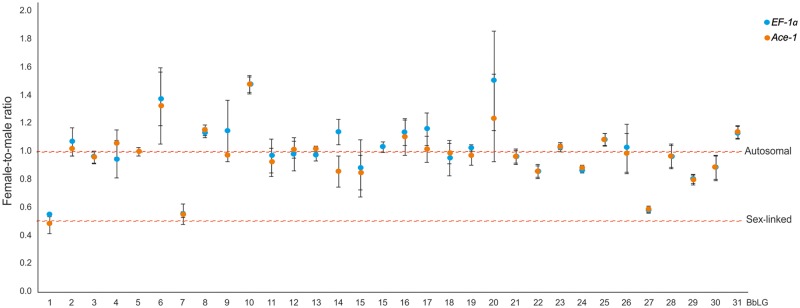
—Screening of marker genes in *Tuta absoluta* by means of qPCR. Blue dots represent the average female-to-male ratio values obtained for each marker using *EF-1a* as the reference gene. Orange dots are the average values for the same variable obtained using *Ace-1* as the reference gene. Whiskers show the SE. Red dashed lines are used to show how each value correlates with 1 (autosomal) and 0.5 (sex-linked) expected female-to-male ratios. Note that most of the data points fluctuate ∼1, except for those corresponding to BbLG1, BbLG7, and BbLG27 which are closer to 0.5 than to 1. BbLG, *Biston betularia* linkage group.

The results of the screening of marker genes in *T. absoluta* are presented in supplementary table S2, Supplementary Material online and figure 3. Markers orthologous to genes of *B.betularia* (Geometridae) LG1 (*ket*), LG7 (*Pix*) and LG27 (*Hsp90*) were sex-linked in this species, with F:M ratios ranging from 0.491 (*ket*) to 0.590 (*Hsp90*), considering the values obtained with both reference genes (*EF-1a* and *Ace-1*). The rest of the markers ranged from 0.800 for *ribosomal protein L4* (marker for BbLG29) to 1.508 for *18–56 protein* (marker for BbLG20), and were considered autosomal. Deviation of markers from the expected F:M value of 1 could be attributed to differences in primer efficiency, which was not corrected in the initial screening.

BbLG1 corresponds to the Z chromosomes in the ancestral karyotype of *n* = 31, while the other two chromosomes (BbLG7 and BbLG27) are autosomes. An extra marker gene was hence considered for further analysis of these autosomes: *Chit* for BbLG7 and *Tw* for BbLG27. Orthologs of all four marker and both reference genes were then amplified and cloned from *P. operculella*, *S. cerealella*, *C. laricella*, *O. arenosella*, *H. pseudospretella*, and *D. daucella*. The partial sequences were deposited in NCBI (accession numbers in supplementary table S1, Supplementary Material online) and used for the design of species-specific primers for qPCR experiments (supplementary table S4, Supplementary Material online).

**Fig. 4.**
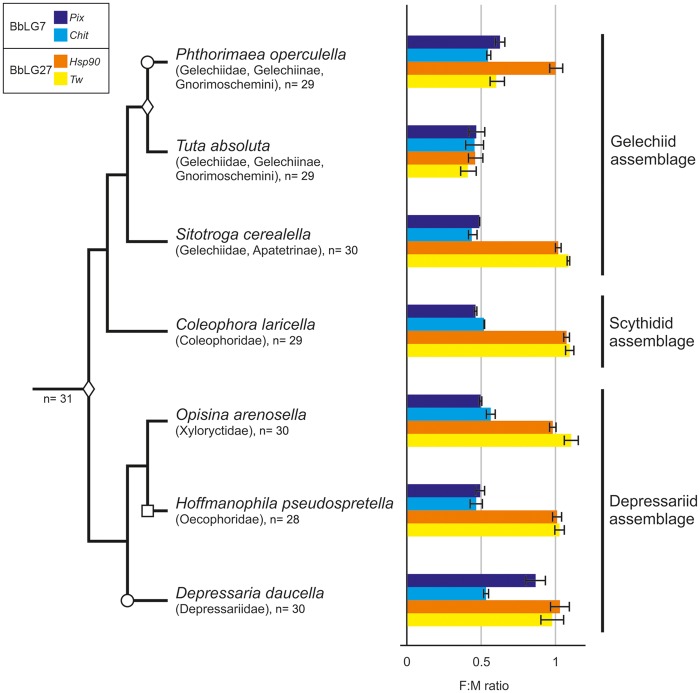
—Phylogenetic relationship between the species analyzed in this study, including a graphic representation of the results obtained using qPCR for the analysis of selected marker genes. Bar charts show the obtained female-to-male ratios (including SEs) of the copy number of the selected marker genes *Pix* and *Chit* for BbLG7, and *Hsp90* and *Tw* for BbLG27, using *EF-1a* as the reference gene. Values close to 0.5 indicate sex-linkage, while values close to 1 indicate autosomal location of the marker. F:M ratio, female-to-male ratio. Note that the decrease in chromosome numbers coincides with sex chromosome–autosome fusions confirmed by qPCR. Diamond, confirmed fusion; circle, translocation or incomplete degeneration of one marker; square, putative fusion suggested by cytogenetic data.

The results in *T. absoluta* and the other gelechioid species are shown in supplementary table S3, Supplementary Material online and summarized in figure 4. The F:M ratio values for both chromosomal markers corresponding to BbLG7 significantly differed from 1 (*P *<* *0.05) but not from 0.5 (*P *>* *0.05) in all species except for *D. daucella*, which suggested that the markers were sex-linked. In *D. daucella*, the F:M ratio of *Chit* was 0. 528 ± 0.021, while for *Pix* it was 0. 861 ± 0.074 (*EF-1a* as the reference gene), which is consistent with sex-linkage of the former and autosomal inheritance of the latter.

For the BbLG27 markers, the F:M ratio statistically differed from 0.5 (*P *<* *0.05) but not from 1 (*P *>* *0.05) in *S. cerealella*, *C. laricella*, *O. arenosella*, *H. pseudospretella*, and *D. daucella*, indicating that the markers had an autosomal location. The opposite situation was observed in *T. absoluta*, meaning that both markers were sex-linked in this species. Interesting results were obtained in *P. operculella*, where *Tw* was sex-linked but *Hsp90* was not (F:M ratios of 0. 578 ± 0. 019 and 0.999 ± 0.047, respectively; *EF-1a* as the reference gene). These findings, together with the discrepancies found for the markers for BbLG7 in *D. daucella*, were corroborated using the second reference gene (*Ace-1*) with a similar outcome (supplementary table S3, Supplementary Material online).

## Discussion

In this study, we analyzed the sex chromosomes of seven species sampled across all three major lineages of the superfamily Gelechioidea (cf. Sohn et al. 2016; for phylogenetic relationships, see fig. 4 and supplementary fig. S1, Supplementary Material online). All species under study have a derived chromosome number compared with the ancestral lepidopteran karyotype of *n* = 31. Our cytogenetic analyses confirmed the expected presence of a large chromosome pair in the karyotypes of *T.absoluta* (Gelechiidae), *C.laricella* (Coleophoridae), and *H.pseudospretella* (Oecophoridae), species with karyotypes reduced to *n* = 29 in the first two and *n* = 28 in the latter (figs. 1*A, B*, *E, F* and 2*D*, *E*). The existence of a conspicuously large chromosome pair was a characteristic feature of the Gelechioidea karyotypes described to date (supplementary table S6, Supplementary Material online) and Ennis (1976) regarded them as autosomal fusion products. The GISH experiments performed in this study, however, confirmed that the largest chromosome pairs are indeed sex chromosomes in *T. absoluta* and *H. pseudospretella* (figs. 1*A* and 2*E*). In *C. laricella*, the W chromosome could not be identified (not shown). Thus, our cytogenetic data suggest that the largest chromosome pair corresponds to sex chromosomes only in some gelechioid species. A similar size difference, that is, the largest chromosome pair being about 1.5–2 times larger than the second largest one in a descending size series, was also observed in other *Coleophora* species (Lukhtanov and Puplesiene 1999) and in *P.operculella* (Gelechiidae) (Bedo 1984) suggesting chromosome fusions. Interspecific differences were observed in the relative size of the sex chromosomes, which were not so conspicuous in species with *n* = 30, namely *S.cerealella* (Gelechiidae), *O.arenosella* (Xylorictidae), and *D.daucella* (Depressaridae) (figs. 1*C, D* and 2*A–C*, *G–I*). A larger chromosome pair, which was not detected in our study, was reported for *S. cerealella* by Lukhtanov and Kuznetsova (1989) based on preparations of metaphase I bivalents from males (supplementary table S6, Supplementary Material online). This inconsistency could be caused by different methods, tissues used for chromosome preparations, and the type of cell division.

To confirm the fusions and identify the synteny blocks involved, we tested selected markers for all chromosomes of the ancestral karyotype with *n* = 31 (Van’t Hof 2013; Ahola et al. 2014) for their sex-linkage in *T. absoluta* by means of qPCR*.* The qPCR results confirmed the sex-linkage of markers located on the Z chromosome in other Lepidoptera (Nguyen et al. 2013; Van’t Hof 2013) and identified synteny blocks homoeologous to *B.betularia* (Geometridae) linkage group (BbLG) 7 and 27 as candidates for fusions (fig. 3). Testing of two markers for each chromosome, namely *Pix* and *Chit* for BbLG7, and *Hsp90* and *Tw* for BbLG27, confirmed their sex-linkage in *T. absoluta* and thus strongly supported fusions of these synteny blocks with the ancestral Z chromosome (supplementary table S3, Supplementary Material online and fig. 4). qPCR analyses of *Pix* and *Chit* in the other species showed a sex-linkage of both markers in all gelechioids but *D. daucella*, in which only *Chit* and not *Pix* was sex-linked (supplementary table S3, Supplementary Material online and fig. 4). Assuming current phylogenetic hypotheses (Heikkilä et al. 2014; Sohn et al. 2016; supplementary fig. S1, Supplementary Material online), the qPCR results suggest that the fusion of the Z chromosome and chromosome homoeologous to BbLG7 [hereinafter F(Z; 7)] occurred in a common ancestor of the superfamily Gelechioidea. Thus, the autosomal location of *Pix* in *D. daucella* most likely points to a secondary translocation of this gene to an unidentified autosome (cf. Nguyen et al. 2013) or the W chromosome (Van’t Hof 2013) or to incomplete degeneration of its W-linked copy. The latter, however, seems unlikely in this case, as the F(Z; 7) fusion occurred ∼100 Ma (Wahlberg et al. 2013). Sex-linkage analyses of *Hsp90* and *Tw* revealed that these markers are autosomal in all species but two representatives of the family Gelechiidae, *T. absoluta* and *P. operculella*, with *Tw* sex-linked in the latter but not *Hsp90* (supplementary table S3, Supplementary Material online and fig. 4). This, together with the autosomal localization of both markers in *S. cerealella*, suggests that the neo-Z chromosome formed by the F(Z; 7) fusion further fused with BbLG27 [hereinafter F(neo-Z; 27)] in a common ancestor of the tribe Gnorimoschemini. However, we cannot exclude the possibility that the F(neo-Z; 27) fusion occurred earlier in the subfamily Gelechiinae (cf. Karsholt et al. 2013). Autosomal linkage of *Hsp90* in *P. operculella* can again be explained by its translocation (see above). However, given the relative young age of the F(neo-Z; 27) fusion, we cannot fully rule out the *Hsp90* allele persisting on the neo-W chromosome. Further research is needed to trace the exact evolutionary origin and level of differentiation of the F(neo-Z; 27) fusion. Moreover, the reduced chromosome number observed in *H. pseudospretella* (see above), the large size of its neo-sex chromosome pair along with the partial differentiation of its W chromosome suggest that another fusion between the F(Z; 7) and an autosome occurred independently in the family Oecophoridae.

Our results hence clearly show that at least two sex chromosome–autosome fusions occurred in the evolution of the diverse superfamily Gelechioidea. This finding further adds to the growing list of derived sex chromosome systems recently identified in various lepidopteran taxa, such as leafrollers of the family Tortricidae (Nguyen et al. 2013; Šíchová et al. 2013; Picq et al. 2018), leaf miners of the family Gracillaridae (Dalíková et al. 2017; Fraïsse et al. 2017), and *Leptidea* wood white (Pieridae) (Šíchová et al. 2015, 2016) and *Danaus* (Nymphalidae) butterflies (Smith et al. 2016; Mongue et al. 2017; Traut et al. 2017). The latter represent yet another case of repeated sex chromosome–autosome fusions, similar to those reported in this study. All these findings illustrate that neo-sex chromosomes are not exceptional in moths and butterflies. Rather, they appear to be relatively common, not only in terms of number of species, as the Tortricidae and Gelechioidea taxa alone comprise together about 17% of the described lepidopteran biodiversity (Beccaloni et al. 2018) but also in the number of independent origins (Nguyen and Carabajal Paladino 2016; cf. Pokorná et al. 2014). This suggests that the paucity of sex chromosome–autosome fusions is not an intrinsic feature of female heterogamety as previously assumed (Pokorná et al. 2014; Pennell et al. 2015).

Lepidoptera possess holokinetic chromosomes, which attach to kinetochore microtubules along most of the chromosomal surface (Wolf 1994). This reduces the risk of formation of dicentric and acentric chromosomes and hence it is expected to facilitate chromosomal rearrangements (Wrensch et al. 1994). Indeed, high variation in chromosome numbers was observed in moths and butterflies (Blackmon et al. 2017). However, this genome instability is confined only to a few lepidopteran taxa (Robinson 1971; Talavera et al. 2013). Comparative genomic studies have revealed that lepidopteran karyotypes are very stable with the modal chromosome number of *n* = 31 being the ancestral one. Furthermore, it has been shown that chromosome fusions are not random in this insect order since independent fusions observed in distant species involve the same small and repeat-rich chromosomes (Van’t Hof 2013; Ahola et al. 2014). Reconstructions of karyotype evolution in several lepidopteran clades with derived sex chromosome systems also show that the first large-scale chromosome rearrangements which differentiated the karyotypes of examined taxa from the ancestral *n* = 31 tend to be sex chromosome–autosome fusions (Nilsson et al. 2008; Nguyen et al. 2013; Šíchová et al. 2013; Dalíková et al. 2017; Mongue et al. 2017). Although the reconstruction of karyotype evolution in a group so diverse as Gelechioidea is challenging due to the scarcity of available data (supplementary table S6, Supplementary Material online), the reduced chromosome number of *n* = 30 in families Gelechiidae, Elachistidae, Xyloryctidae, and Depressariidae suggests that the F(Z; 7) fusion occurred early in the karyotype evolution of gelechioids.

This propensity of lepidopteran sex chromosomes for fusions could shed light on the evolutionary forces driving chromosomal change. The higher rate of sex chromosome–autosome fusions in XX/XY than in WZ/ZZ systems observed in vertebrates (Pokorná et al. 2014; Pennell et al. 2015) led to the conclusion that fusions must be driven by two or more evolutionary forces (Pennell et al. 2015; Kirkpatrick 2017). A simpler explanation for the higher rate of Y-autosome fusions in vertebrates, random genetic drift (Kirkpatrick 2017), was dismissed due to the lack of multiple sex chromosomes in female heterogametic groups (Pennell et al. 2015; Kirkpatrick 2017). Genetic drift, however, can be invoked to explain the high incidence of neo-sex chromosomes in Lepidoptera. In such case, the same pattern observed in vertebrates (a higher incidence of W–autosome than Z–autosome fusions) is expected for lepidopteran multiple sex chromosome systems. However, the W–autosome and Z–autosome fusions resulting in multiple sex chromosome constitutions WZ_1_Z_2_ and W_1_W_2_Z, respectively, observed so far in Lepidoptera are tied (Traut et al. 2007; Šíchová et al. 2015, 2016; Smith et al. 2016). Furthermore, many of the other recently reported neo-sex chromosomes systems are not informative as males and females exhibit the same chromosome number (Nguyen et al. 2013; Dalíková et al. 2017; Fraïsse et al. 2017; Mongue et al. 2017; this study). Available data thus do not allow us to evaluate the role of genetic drift in sex chromosome–autosome fusions in Lepidoptera.

Chromosome rearrangements such as fusions or inversions affect linkage relationships and thus can play an important role in adaptation and speciation (Yeaman 2013; Charlesworth 2015; Ortiz-Barrientos et al. 2016). In leafrollers of the family Tortricidae, Nguyen et al. (2013) reported the fusion of the Z chromosome with an autosome homoeologous to BbLG15. This chromosome is enriched in genes involved in detoxification and regulated absorption of plant secondary metabolites, namely esterases and ABC transporters, which are crucial for the performance of lepidopteran larvae on their host plants. The fusion thus linked these performance genes together with sex-linked female preference or host-independent isolation genes, which can facilitate adaptation and speciation in the presence of gene flow (Matsubayashi et al. 2010). Furthermore, it was hypothesized that the neo-Z-linked performance genes got amplified to make up for their nonrecombining and thus gradually degenerating maternally inherited gametologues (Nguyen et al. 2013). Following functional divergence of the new performance gene copies supposedly contributed to adaptation to new hosts which could eventually result in the formation of new species (cf. Li et al. 2003). Interestingly, BbLG7, which is involved in the F(Z; 7) fusion shared by all gelechioids, comprises the largest cluster of UDP-glycosyltransferases (UGTs). Enzymes encoded by the UGT gene family catalyze the glycosylation of small lipophilic compounds, turning them into water-soluble and thus more easily excreted products (Ahn et al. 2012). Although UGTs have been considerably understudied compared with other detoxification families, evidence supporting their role in detoxification of plant secondary metabolites and insecticides in Lepidoptera has been growing (Ahn et al. 2011; Wouters et al. 2014; Krempl et al. 2016; Li et al. 2017). Therefore, we hypothesize that the sex chromosome–autosome fusions may indeed contribute to ecological specialization and speciation in moths.

Sex chromosome turnover has been shown to predate, so far, two large lepidopteran radiations, Tortricidae and Gelechioidea (Nguyen et al. 2013; this study). The F(Z; 7) fusion observed in gelechioids fits well the scenario drawn by Nguyen et al. (2013) and the enrichment in performance genes of the autosomes involved in fusions in both lineages points to more general aspects of the lepidopteran karyotype evolution. The superfamily Gelechioidea provides an opportunity to test the hypothesis on the role of neo-sex chromosomes in the speciation of Lepidoptera, as sister lineages with and without neo-sex chromosomes of different age can be examined in parallel, along with their diversification rates.

## Supplementary Material

Supplementary data are available at *Genome Biology and Evolution* online.

## Supplementary Material

Supplementary Data
