# Supplementary material for: Sex Chromosome Turnover in Moths of the Diverse Superfamily Gelechioidea
Source: Genome Biol Evol. 2019 Apr 8;11(4):1307–19. doi: 10.1093/gbe/evz075 (PMC6486803; doi:10.1093/gbe/evz075)
Supplement: Supplementary Data [file evz075_supp.docx]

**Supplementary Table S1**. List of partial sequences of gelechioid genes obtained in this study.

| Species | Gene ^a^ | Primers ^b^ | GeneBank accession number |
| --- | --- | --- | --- |
| *Phthorimaea operculella* | *Hsp90* | *Hsp90* F01 + R01 | MG265668 |
|  | *Ace-1* | *AceI* F01 + R01 | MG265690 |
|  | *Chit* | *Chit* F01 + R01 | MG265651 |
|  | *EF-1a* | *EF1a* F01 + R01 | MG265660 |
|  | *Pix* | *Pix* F01 + R01 | MG265675 |
|  | *Tw* | *Tw* F01 + R01 | MG265684 |
| *Sitotroga cerealella* | *Hsp90* | *Hsp90* F01 + R01 | MG265669 |
|  | *Chit* | *Chit* F01 + R01 | MG265658 |
|  | *EF-1a* | *EF1a* F01 + R01 | MG265659 |
|  | *Pix* | *Pix* F01 + R01 | MG265680 |
|  | *Tw* | *Tw* F01 + R01 | MG265683 |
| *Coleophora laricella* | *Hsp90* | *Hsp90* F01 + R01 | MG265670 |
|  | *Chit* | *Chit* F01 + R01 | MG265657 |
|  | *COI* | Hebert *et al.* 2004 | MG265692 |
|  | *EF-1a* | *EF1a* F01 + R01 | MG265667 |
|  | *Pix* | *Pix* F01 + R01 | MG265679 |
|  | *Tw* | *Tw* F01 + R01 | MG265687 |
| *Opisina arenosella* | *Hsp90* | *Hsp90* F01 + R02 | MG265673 |
|  | *Chit* | *Chit* F01 + R01 | MG265652 |
|  | *EF-1a* | *EF1a* F01 + R01 | MG265661 |
|  | *Pix* | *Pix* F02 + R01 | MG265681 |
|  | *Tw* | *Tw* F02 + R01 | MG265688 |
| *Hofmannophila pseudospretella* | *Hsp90* | *Hsp90* F01 + R01 | MG265672 |
|  | *Chit* | *Chit* F01 + R01 | MG265654 |
|  | *COI* | Hebert *et al.* 2004 | MG265693 |
|  | *EF-1a* | *EF1a* F01 + R01 | MG265664 |
|  | *Pix* | *Pix* F02 + R01 | MG265682 |
|  | *Tw* | *Tw* F01 + R01 | MG265685 |
| *Depressaria daucella* | *Hsp90* | *Hsp90* F01 + R02 | MG265674 |
|  | *Ace-1* | *AceI* F01 + R01 | MG265689 |
|  | *Chit* | *Chit* F01 + R01 | MG265655, MG265656 |
|  | *COI* | Hebert *et al.* 2004 | MG265694 |
|  | *EF-1a* | *EF1a* F01 + R01 | MG265665, MG265666 |
|  | *Pix* | *Pix* F01 + R01 | MG265678 |
|  | *Tw* | *Tw* F01 + R01 | MG265686 |

^a^ *COI*, *cytochrome c oxidase subunit I*; for full names of the other genes, see Supplementary Table S6.

^b^ For sequences, see Supplementary Table S5.

**Supplementary Table S2**. List of primers and results of quantitative PCR experiments carried out for the screening of sex-linked genes in *Tuta absoluta*.

| BbLG | Marker gene | Forward primer | Reverse primer | Reference gene: *EF-1a* | | Reference gene: *Ace-1* | |
| --- | --- | --- | --- | --- | --- | --- | --- |
|  |  |  |  | F:M ratio | S.E. | F:M ratio | S.E. |
| 1 | *kettin* | AAGGAGGATTCGCACACTTC | CGTGTACGGTAACGGACTTT | 0.552 | 0.014 | 0.491 | 0.070 |
| 2 | *ribosomal protein L23* | GGTAATCCGGCAGCGGAAA | ATACGGGGCCACAAATCGG | 1.071 | 0.099 | 1.023 | 0.053 |
| 3 | *ribosomal protein L13A* | ATGATCCCACACAAGACGGA | AGACGCGCAAAGCGG | 0.960 | 0.039 | 0.961 | 0.044 |
| 4 | *eukaryotic translation initiation factor 3 subunit H* | CAACCTGAAGATCGGCTACG | CTTCTAGCACTGAAGCGGTC | 0.946 | 0.134 | 1.058 | 0.098 |
| 5 | *Elongation factor 1 alpha* | CAGTGTGACAGTCGAGTACG | CAACGTCAAGAACGTTTCCG | N/A | N/A | 1.002 | 0.029 |
| 6 | *Ras3 protein* | ATCGTTGAAAGTGGATTGCG | AACGATTGAGGACAGCTACC | 1.377 | 0.190 | 1.325 | 0.272 |
| 7 | *pixie ATP-binding cassette sub-family E member 1* | ACGCAAAACGAACAGGATTC | AGTTGTTAGGATCTCTGCGG | 0.553 | 0.020 | 0.557 | 0.072 |
| 8 | *Adaptor Protein complex 2 alpha* | AAGGAGCTAGCCAACATACG | AGAGGAAAGTAGGTTCACCG | 1.136 | 0.036 | 1.155 | 0.037 |
| 9 | *ribosomal protein S14* | CCACTGGAGGAAACAAGACA | CTAACAGTTTACAGCCTGCG | 1.148 | 0.219 | 0.973 | 0.008 |
| 10 | *ribosomal protein L10* | CTATGTGTGCACTTGGTGTC | CAGCGCACGATAACATTTTG | 1.477 | 0.066 | 1.479 | 0.056 |
| 11 | *G protein pathway suppressor 1* | TAGCATGGATGTCCGTCTTT | TGATGGCTGCTGCATTTAAC | 0.972 | 0.121 | 0.927 | 0.101 |
| 12 | *transcription factor TFIIE beta subunit* | ATAAAACAATGGCAGGCAGC | TATTCCCTACATCAAGCTGGT | 0.983 | 0.118 | 1.016 | 0.062 |
| 13 | *troponin T* | CTCTCTGTCCATCCGCATCA | GTCGTAGTCCTGTCGCTTTTG | 0.981 | 0.044 | 1.021 | 0.019 |
| 14 | *ribosomal protein S3* | TATCTGAGGGACTCTGCCTG | CTACGAGGACCCAAAGTGTG | 1.142 | 0.090 | 0.861 | 0.112 |
| 15 | *minichromosome maintenance complex component 7* | GATTGGGCTTGTCCTGTATC | GGTATAATGACGTGCCTGAAC | 0.884 | 0.205 | 0.852 | 0.122 |
| 15 | *Acetylcholinesterase 1* | TGGTAATCGCCCACCATTTT | GCCTAAGGAAGAGAACGTGG | 1.034 | 0.039 | N/A | N/A |
| 16 | *helicase* | CAAGTGATGATGTTCTCCGC | TCCAATACATCCAGAAGCTCG | 1.137 | 0.089 | 1.105 | 0.130 |
| 17 | *cathepsin B* | GGGACTAAGCACTTCCACTT | ATCTCGTATGGACGACAACC | 1.163 | 0.115 | 1.018 | 0.092 |
| 18 | *arylalkylamine N-acetyltransferase* | GAGAGACTTGGCTACAAGAG | TCTTTGATGTAAACTCTAGCTTC | 0.956 | 0.124 | 0.990 | 0.073 |
| 19 | *ATP synthase* | AGATGTCCGACAAAATGGGC | CAGACAGACCGACGTTGATG | 1.028 | 0.024 | 0.972 | 0.067 |
| 20 | *18-56 protein* | GGAAGGATTCCGACCGTATT | TCTTGTCCATCGGTTTGACC | 1.508 | 0.354 | 1.242 | 0.311 |
| 21 | *furin-like protease 1* | ATATTCGACAACCACTACCAC | GGAAGTCTCTTTTCTTCCTTGA | 0.964 | 0.056 | 0.965 | 0.047 |
| 22 | *ribosomal protein S4* | GTAACCTGTGCATGATCACC | CTAGTAGCGAAGGTGTGCC | 0.860 | 0.051 | 0.860 | 0.041 |
| 23 | *polycomb protein Scm* | AAATGGAGCTGAAATGGCAC | GTCAAATGCACCTCTCCAAC | 1.033 | 0.032 | 1.034 | 0.020 |
| 24 | *mitogen-activated protein kinase kinase kinase 5* | GATACGAGCCGTAGTAAGCC | ACAAGGCCAAGAGACTCAAC | 0.870 | 0.018 | 0.885 | 0.019 |
| 25 | *26S protease regulatory subunit 6A-B* | TCATTGAATTGCTGGACGTA | CCAACTAAATCTCCAGGCTT | 1.027 | 0.170 | 0.988 | 0.142 |
| 26 | *26S proteasome regulatory ATPase subunit 10B* | CCCTAATGGAGCTACTCAACC | GTTCATTGGGCAGCGGTAT | 0.902 | 0.195 | 0.792 | 0.245 |
| 27 | *90-kDa heat shock protein* | AAGTTCGAGGTACTCAGGGA | TGAAGGTCAACTAGAGTTCCG | 0.582 | 0.020 | 0.590 | 0.022 |
| 28 | *protein kinase C alpha binding protein* | AGTTCATTGAGCTTGGCAAC | AACTACAACAAACTCCACGC | 0.970 | 0.087 | 0.968 | 0.076 |
| 29 | *ribosomal protein L4* | CACATCATCGAGAAAATCCCC | CTTGAGCACGTCAGACCAG | 0.807 | 0.035 | 0.800 | 0.036 |
| 30 | *acetyl_Coa carboxylase* | TCACCTCAATCTCAGCATAGG | GACGAGGTGGACAAGTACC | 0.885 | 0.090 | 0.886 | 0.082 |
| 31 | *H/ACA ribonucleoprotein complex subunit 4-like* | TTTGACCAGTCTCGTAGCTC | CAAGTCTGGCTTCATCAACG | 1.138 | 0.041 | 1.140 | 0.047 |

BbLG, *Biston betularia* linkage group; F:M, female-to-male ratio; S.E., standard error; N/A, non-applicable.

**Supplementary Table S3**. Results of quantitative PCR analyses of gene dose for the identification of sex-linked synteny blocks in Gelechioidea.

|  |  |  |  |  | Ct values (target/reference) | | | | Female-to-male ratio | | | | | *P* | |
| --- | --- | --- | --- | --- | --- | --- | --- | --- | --- | --- | --- | --- | --- | --- | --- |
| Species | Target ^a^ | *E*  target | *E*  reference | Sex | Sample  I | Sample  II | Sample  III | Mean | Sample  I | Sample  II | Sample  III | Mean | S.E. | H_0_  F:M=1 ^b^ | H_0_  F:M=0.5 ^c^ |
| *Phthorimaea*  *operculella* | *Chit* ^d^ | 0.897 | 0.849 (*EF-1a*) | F | 20.147/19.170 | 20.787/19.860 | 20.360/19.550 | - | 0.525 | 0.533 | 0.579 | 0.546 | 0.017 | **0.001** | 0.112 |
|  |  |  |  | M | 19.570/19.567 | 19.523/19.520 | 19.720/19.870 | 19.604/19.652 |  |  |  |  |  |  |  |
|  | *Hsp90* ^d^ | 0.924 | 0.888 (*Ace-1*) | F | 19.887/10.207 | 20.007/20.270 | 19.603/20.060 | - | 1.049 | 1.010 | 1.150 | 1.070 | 0.042 | 0.238 | **0.005** |
|  |  |  |  | M | 19.957/20.173 | 19.890/20.233 | 20.017/20.197 | 19.954/20.201 |  |  |  |  |  |  |  |
|  | *Hsp90* ^d^ | 0.924 | 0.849 (*EF-1a*) | F | 19.523/19.170 | 20.320/19.860 | 19.777/19.550 | - | 1.006 | 0.913 | 1.077 | 0.999 | 0.047 | 0.978 | **0.009** |
|  |  |  |  | M | 19.960/19.567 | 19.843/19.520 | 20.153/19.870 | 19.986/19.652 |  |  |  |  |  |  |  |
|  | *Pix* ^d^ | 0.865 | 0.849 (*EF-1a*) | F | 20.760/19.897 | 20.943/19.860 | 20.503/19.687 | - | 0.642 | 0.560 | 0.662 | 0.622 | 0.031 | **0.007** | 0.060 |
|  |  |  |  | M | 20.127/19.970 | 19.977/19.790 | 20.683/20.577 | 20.262/20.112 |  |  |  |  |  |  |  |
|  | *Tw* ^e^ | 0.698 | 0.888 (*Ace-1*) | F | 23.223/20.207 | 23.570/20.270 | 23.147/20.060 | - | 0.544 | 0.472 | 0.516 | 0.511 | 0.021 | **0.002** | 0.662 |
|  |  |  |  | M | 22.167/20.173 | 21.887/20.233 | 22.150/20.197 | 22.068/20.201 |  |  |  |  |  |  |  |
|  | *Tw* ^e^ | 0.698 | 0.849 (*EF-1a*) | F | 23.710/20.230 | 24.060/20.373 | 23.777/20.133 | - | 0.615 | 0.558 | 0.559 | 0.578 | 0.019 | **0.002** | 0.054 |
|  |  |  |  | M | 22.923/20.227 | 22.683/20.130 | 22.847/20.400 | 22.818/20.252 |  |  |  |  |  |  |  |
| *Sitotroga*  *cerealella* | *Chit* ^d^ | 0.718 | 0.826 (*EF-1a*) | F | 22.197/19.897 | 22.573/20.600 | 22.407/20.107 | - | 0.402 | 0.501 | 0.408 | 0.437 | 0.032 | **0.003** | 0.190 |
|  |  |  |  | M | 21.607/20.910 | 20.907/20.527 | 21.257/20.253 | 21.257/20.563 |  |  |  |  |  |  |  |
|  | *Hsp90* ^d^ | 0.729 | 0.826 (*EF-1a*) | F | 20.393/21.977 | 20.180/21.660 | 20.183/21.753 | - | 1.040 | 0.970 | 1.020 | 1.013 | 0.022 | 0.612 | **0.002** |
|  |  |  |  | M | 20.180/21.720 | 20.197/21.693 | 20.310/21.850 | 20.229/21.754 |  |  |  |  |  |  |  |
|  | *Pix* ^f^ | 0.756 | 0.935 (*EF-1a*) | F | 20.397/19.397 | 20.677/19.713 | 20.520/19.583 | - | 0.480 | 0.477 | 0.491 | 0.483 | 0.004 | **0.000** | 0.060 |
|  |  |  |  | M | 19.550/19.737 | 19.357/19.543 | 19.583/19.657 | 19.497/19.646 |  |  |  |  |  |  |  |
|  | *Tw* ^d^ | 0.721 | 0.826 (*EF-1a*) | F | 19.517/21.423 | 19.693/21.633 | 19.643/21.727 | - | 0.967 | 0.997 | 1.084 | 1.016 | 0.035 | 0.693 | **0.005** |
|  |  |  |  | M | 19.790/21.733 | 19.477/21.423 | 19.860/21.800 | 19.709/21.652 |  |  |  |  |  |  |  |
| *Tuta*  *absoluta* | *AceI* ^d^ | 1.036 | 0.990 (*EF-1a*) | F | 22.307/24.077 | 18.947/20.977 | 19.177/21.363 | - | 0.807 | 1.040 | 1.155 | 1.000 | 0.102 | 0.988 | **0.039** |
|  |  |  |  | M | 21.257/23.100 | 22.780/25.123 | 21.993/24.020 | 22.010/24.081 |  |  |  |  |  |  |  |
|  | *Chit* ^d^ | 1.021 | 0.976 (*EF-1a*) | F | 21.097/21.045 | 22.470/22.083 | 21.540/21.843 | - | 0.441 | 0.340 | 0.556 | 0.445 | 0.062 | **0.012** | 0.473 |
|  |  |  |  | M | 21.173/22.283 | 21.713/22.810 | 23.473/24.823 | 22.120/23.306 |  |  |  |  |  |  |  |
|  | *Hsp90* ^h^ | 1.028 | 0.976 (*EF-1a*) | F | 26.973/24.583 | 27.200/25.050 | 25.385/22.583 | - | 0.458 | 0.537 | 0.361 | 0.452 | 0.051 | **0.008** | 0.444 |
|  |  |  |  | M | 26.900/25.517 | 27.287/25.890 | 25.247/24.237 | 27.093/25.703 |  |  |  |  |  |  |  |
|  | *ket* ^h^ | 0.999 | 0.974 (*Ace-1*) | F | 19.827/18.023 | 20.497/18.737 | 21.770/20.680 | - | 0.399 | 0.417 | 0.677 | 0.498 | 0.090 | **0.030** | 0.983 |
|  |  |  |  | M | 19.853/19.427 | 19.173/18.910 | 19.077/18.443 | 19.367/18.927 |  |  |  |  |  |  |  |
|  | *Pix* ^g^ | 0.934 | 0.976 (*EF-1a*) | F | 21.380/21.045 | 22.723/22.083 | 21.837/21.843 | - | 0.441 | 0.369 | 0.562 | 0.457 | 0.056 | **0.011** | 0.526 |
|  |  |  |  | M | 21.390/22.283 | 22.120/22.810 | 23.907/24.823 | 22.472/23.306 |  |  |  |  |  |  |  |
|  | *Tw* ^i^ | 0.907 | 0.976 (*EF-1a*) | F | 24.313/24.300 | 24.517/25.010 | 21.823/21.523 | - | 0.368 | 0.507 | 0.334 | 0.403 | 0.053 | **0.008** | 0.209 |
|  |  |  |  | M | 22.797/23.833 | 23.830/25.500 | 22.040/23.700 | 22.889/24.344 |  |  |  |  |  |  |  |
| *Coleophora*  *laricella* | *Chit* ^j^ | 1.015 | 1.034 (*EF-1a*) | F | 21.440/20.310 | 21.910/20.707 | 21.520/20.360 | - | 0.528 | 0.504 | 0.517 | 0.516 | 0.007 | **0.000** | 0.149 |
|  |  |  |  | M | 20.337/20.037 | 20.173/20.093 | 20.863/20.590 | 20.458/20.240 |  |  |  |  |  |  |  |
|  | *Hsp90* ^d^ | 0.956 | 0.926 (*EF-1a*) | F | 22.307/22.450 | 22.693/22.957 | 22.613/22.817 | - | 1.030 | 1.110 | 1.070 | 1.070 | 0.022 | 0.090 | **0.002** |
|  |  |  |  | M | 22.320/22.437 | 21.597/21.560 | 22.713/22.913 | 22.210/22.303 |  |  |  |  |  |  |  |
|  | *Pix* ^j^ | 1.043 | 1.034 (*EF-1a*) | F | 22.253/20.847 | 23.103/21.820 | 21.943/20.623 | - | 0.435 | 0.473 | 0.463 | 0.457 | 0.011 | **0.000** | 0.064 |
|  |  |  |  | M | 21.350/21.113 | 21.367/21.163 | 21.387/21.107 | 21.368/21.128 |  |  |  |  |  |  |  |
|  | *Tw* ^d^ | 0.842 | 0.926 (*EF-1a*) | F | 21.817/21.520 | 22.303/22.107 | 22.150/21.980 | - | 1.020 | 1.120 | 1.130 | 1.090 | 0.033 | 0.114 | **0.003** |
|  |  |  |  | M | 21.850/21.393 | 22.003/21.683 | 22.423/22.147 | 22.092/21.741 |  |  |  |  |  |  |  |
| *Opisina*  *arenosella* | *Chit* ^f^ | 1.071 | 0.803 (*EF-1a*) | F | 21.353/20.660 | 21.440/20.393 | 21.600/20.793 | - | 0.619 | 0.497 | 0.560 | 0.559 | 0.035 | **0.006** | 0.239 |
|  |  |  |  | M | 20.623/20.400 | 19.803/19.607 | 20.650/20.727 | 20.359/20.244 |  |  |  |  |  |  |  |
|  | *Hsp90* ^d^ | 0.701 | 0.924 (*EF-1a*) | F | 24.573/21.083 | 24.280/20.853 | 25.12/21.417 | - | 0.999 | 1.004 | 0.929 | 0.977 | 0.024 | 0.447 | **0.003** |
|  |  |  |  | M | 25.047/21.330 | 23.610/20.270 | 24.687/21.350 | 24.448/20.983 |  |  |  |  |  |  |  |
|  | *Pix* ^f^ | 1.007 | 0.803 (*EF-1a*) | F | 20.967/20.110 | 20.427/19.360 | 20.980/20.120 | - | 0.503 | 0.471 | 0.501 | 0.492 | 0.010 | **0.000** | 0.508 |
|  |  |  |  | M | 19.493/19.423 | 19.187/19.123 | 19.447/19.640 | 19.376/19.396 |  |  |  |  |  |  |  |
|  | *Tw* ^d^ | 0.791 | 0.924 (*EF-1a*) | F | 21.013/20.623 | 20.650/20.343 | 21.160/20.990 | - | 1.029 | 1.077 | 1.201 | 1.102 | 0.051 | 0.184 | **0.007** |
|  |  |  |  | M | 21.283/20.800 | 20.653/20.260 | 21.133/20.707 | 21.023/20.589 |  |  |  |  |  |  |  |
| *Hofmannophila*  *pseudospretella* | *Chit* ^d^ | 0.974 | 0.884 (*EF-1a*) | F | 22.233/20.477 | 20.823/19.480 | 20.420/19.003 | - | 0.371 | 0.514 | 0.500 | 0.462 | 0.046 | **0.007** | 0.491 |
|  |  |  |  | M | 20.193/19.857 | 19.850/19.207 | 19.947/19.863 | 19.997/19.642 |  |  |  |  |  |  |  |
|  | *Hsp90* ^d^ | 0.843 | 0.884 (*EF-1a*) | F | 20.417/20.410 | 19.523/19.650 | 19.510/19.447 | - | 1.000 | 1.067 | 0.946 | 1.004 | 0.046 | 0.917 | **0.005** |
|  |  |  |  | M | 20.170/20.257 | 19.310/19.363 | 19.520/19.473 | 19.333/19.364 |  |  |  |  |  |  |  |
|  | *Pix* ^k^ | 0.946 | 0.873 (*EF-1a*) | F | 21.507/20.303 | 22.930/21.417 | 20.440/19.007 | - | 0.546 | 0.426 | 0.492 | 0.488 | 0.035 | **0.005** | 0.767 |
|  |  |  |  | M | 20.473/20.093 | 20.743/20.673 | 19.730/19.243 | 20.316/20.003 |  |  |  |  |  |  |  |
|  | *Tw* ^d^ | 0.852 | 0.884 (*EF-1a*) | F | 20.677/20.867 | 20.337/20.470 | 19.603/20.067 | - | 1.096 | 1.051 | 1.279 | 1.142 | 0.070 | 0.179 | **0.012** |
|  |  |  |  | M | 21.113/20.917 | 19.907/20.010 | 19.403/19.677 | 20.141/20.201 |  |  |  |  |  |  |  |
| *Depressaria*  *daucella* | *Chit* ^i^ | 0.982 | 0.952 (*Ace-1*) | F | 21.110/20.550 | 20.793/20.143 | 20.987/20.230 | - | 0.523 | 0.495 | 0.460 | 0.493 | 0.018 | **0.001** | 0.736 |
|  |  |  |  | M | 20.253/20.660 | 19.885/20.133 | 19.627/20.113 | 19.922/20.302 |  |  |  |  |  |  |  |
|  | *Chit* ^i^ | 0.982 | 0.914 (*EF-1a*) | F | 21.107/20.287 | 21.010/20.123 | 21.133/20.460 | - | 0.518 | 0.497 | 0.569 | 0.528 | 0.021 | **0.002** | 0.319 |
|  |  |  |  | M | 20.527/20.983 | 19.923/19.783 | 19.787/19.887 | 20.079/20.218 |  |  |  |  |  |  |  |
|  | *Hsp90* ^d^ | 0.872 | 0.998 (*EF-1a*) | F | 28.357/19.573 | 28.170/19.590 | 28.087/19.663 | - | 0.904 | 1.028 | 1.139 | 1.024 | 0.068 | 0.762 | **0.016** |
|  |  |  |  | M | 29.203/20.597 | 28.240/19.670 | 28.427/19.617 | 28.623/19.961 |  |  |  |  |  |  |  |
|  | *Pix* ^i^ | 0.893 | 0.952 (*Ace-1*) | F | 19.527/19.927 | 19.753/20.253 | 20.203/20.260 | - | 0.886 | 0.954 | 0.719 | 0.853 | 0.070 | 0.169 | **0.037** |
|  |  |  |  | M | 20.417/20.770 | 19.117/20.010 | 19.350/19.833 | 19.628/20.204 |  |  |  |  |  |  |  |
|  | *Pix* ^i^ | 0.893 | 0.914 (*EF-1a*) | F | 19.913/20.287 | 19.683/20.123 | 20.480/20.460 | - | 0.915 | 0.953 | 0.713 | 0.861 | 0.074 | 0.202 | **0.040** |
|  |  |  |  | M | 20.083/20.983 | 19.493/19.783 | 19.537/19.887 | 19.704/20.218 |  |  |  |  |  |  |  |
|  | *Tw* ^d^ | 0.883 | 0.998 (*EF-1a*) | F | 21.533/19.863 | 21.367/20.027 | 21.090/19.880 | - | 0.814 | 1.013 | 1.090 | 0.972 | 0.082 | 0.767 | **0.029** |
|  |  |  |  | M | 20.963/19.573 | 21.160/19.727 | 20.873/19.717 | 20.999/19.672 |  |  |  |  |  |  |  |

The reference gene for each experiment is specified in parentheses. Statistically significant differences are indicated in bold. Ct, threshold cycle; *E*, amplification efficiency of the primers; S.E., standard error; *P*, probability; H_0_, null hypothesis; F, female; M, male; F:M, female-to-male ratio.

^a^ For full names, see Table S5

^b^ Autosomal linkage

^c^ Sex-linkage

^d^ Xceed qPCR SG 2x Mix Lo-Rox (IAB, Prague, Czech Republic), 10 µl reaction, 0.4 µM primers target and reference genes

^e^ Xceed qPCR SG 2x Mix Lo-Rox, 10 µl reaction, 0.8 µM primers target gene, 0.4 µM primers reference gene

^f^ Top-Bio qPCR 2x SYBR Master Mix (Top-Bio, Prague, Czech Republic), 20 µl reaction, 1.25 µM primers target and reference genes

^g^ SYBR Premix Ex *Taq* II (Perfect Real Time) (TaKaRa, Otsu, Japan), 25 µl reaction, 0.3 µM primers target gene, 0.2 µM primers reference gene

^h^ SYBR Premix Ex *Taq* II (Perfect Real Time), 25 µl reaction, 0.2 µM primers target and reference genes

^i^ Xceed qPCR SG 2x Mix Lo-Rox, 20 µl reaction, 0.2 µM primers target and reference genes

^j^ SYBR Premix Ex *Taq* II (Perfect Real Time), 25 µl reaction, 0.4 µM primers target and reference genes

^k^ Top-Bio qPCR 2x SYBR Master Mix, 20 µl reaction, 1 µM primers target and reference genes

For the SYBR Premix Ex *Taq* II (Perfect Real Time) and Top-Bio qPCR 2x SYBR Master Mix, the cycling conditions included an initial denaturation at 95 °C for 3 min, 45 cycles of 94 °C for 30 s, 60 °C for 30 s, 72 °C for 30 s, a final denaturation of 95 °C for 15 s, and then an increase of temperature from 65 to 95 °C with increments of 0.5 °C for 5 s for the generation of melting curves. For the Xceed qPCR SG 2x Mix Lo-Rox, the 72 °C step was removed, and the 60 °C step was extended for a total of 45 s.

**Supplementary Table S4**. List of primers used for quantitative PCR.

| Species | Gene ^a^ | Forward primer | Reverse primer |
| --- | --- | --- | --- |
| *Phthorimaea operculella* | *Hsp90* | TCTACAAGACGGTTGGACAC | AGTCTCTGTCACCAAGGAAG |
|  | *Ace-1* | ACGCGTTGGATAAAATGGTG | TAAGACACCTGTCCATGACG |
|  | *Chit* | AGTTTCCAGTAGACCGAGTG | GCCCAAGGATCGTGAATAGA |
|  | *EF-1a* | CCTTCTCTTTGATTTCGGCG | CAAGAACGTTTCCGTCAAGG |
|  | *Pix* | TTCGCGACCGTGAGATCG | GCTTCACGTCGAGATACGAG |
|  | *Tw* | GAAGATGGCACACTCATCAC | CGTCGGGTTTGTCATAAGC |
| *Sitotroga cerealella* | *Hsp90* | CTACCACACATCTGCCTCAG | ACTCATCAATAGGCTCCGTC |
|  | *Chit* | GAGAAAGTTCCCAGTTGACC | ACACTTTGAAAGGTTCCCG |
|  | *EF-1a* | TTGAAACCAACGTTGTCACC | GCAGGACGTCTACAAAATCG |
|  | *Pix* | ATGACTTTAGCTGCGACCAG | TGACGTGATGAAGCCTATGA |
|  | *Tw* | CAAATGTAACGAAATGGGCG | TGGATTGTGGGCTTTTCAAC |
| *Tuta absoluta* | *Hsp90* | AAGTTCGAGGTACTCAGGGA | TGAAGGTCAACTAGAGTTCCG |
|  | *Ace-1* | TGGTAATCGCCCACCATTTT | GCCTAAGGAAGAGAACGTGG |
|  | *Chit* | GATTTATTCCGATTTGCGGTG | ATGGTTCATTCCACGATGAC |
|  | *EF-1a* | CAGTGTGACAGTCGAGTACG | CAACGTCAAGAACGTTTCCG |
|  | *ket* | AAGGAGGATTCGCACACTTC | CGTGTACGGTAACGGACTTT |
|  | *Pix* | ACGCAAAACGAACAGGATTC | AGTTGTTAGGATCTCTGCGG |
|  | *Tw* | AAAGCTAGCCCAAAACCAAC | GCATTGAGCTCTCCAAGTTC |
| *Coleophora laricella* | *Hsp90* | CAGATGAGGGAGTATGACGG | CTCCACCAATCTGTTCGAGA |
|  | *Chit* | AAGTTACGAACTGACCTCGG | TCTCTTTTGGTCTCCAAGCG |
|  | *EF-1a* | GAAGGACTCTACACACAGGG | CCTGGACAAATCTCAAATGGG |
|  | *Pix* | TACTAAACGCTGTTCGGAGTC | ATGAAGCCAATGAAGATTGAGG |
|  | *Tw* | AGAAAGCAATAAAAGAAAGTAGTCG | CTACACCTTCACCGTGTTTG |
| *Opisina arenosella* | *Hsp90* | CGTGGAGAACGATCTTGGTA | TTTGGGGTGGGTTTCTACTC |
|  | *Chit* | TCCTGTTCCAAATCTGACCC | CAAGGGTCGTGGATAGAGAC |
|  | *EF-1a* | GTCCGTAAAAGAGTTGCGTC | TGATTTCAGCGAGTTTGCAG |
|  | *Pix* | TTCCAGCCAACATTCTGATG | GGGTGACTTCTCTCTACGTG |
|  | *Tw* | GAAGATGGCACACTCATCAC | GCCGTCGGATTAGTCAAAAG |
| *Hofmannophila pseudospretella* | *Hsp90* | GTTCCTTCTCAACCATCAGC | CTTTCACCATCAGAGCCGA |
|  | *Chit* | GATTCATTCCAATTTGCGGC | AAACCCCTCTGAGGTTTCTG |
|  | *EF-1a* | AGGACGTATACAAAATCGGC | GTTCTTGACGTTGAAACCG |
|  | *Pix* | TTATTACTTTGGCGGCTACC | ATGGATCAAGAGATCCAGAAC |
|  | *Tw* | CCGCTGTCACGTCAAAAATG | TGACTTTGCAGCCCATTAATAC |
| *Depressaria daucella* | *Hsp90* | GGAGTGCTTCTTCACGACTT | CGTGACAGTCCACTCTAAACA |
|  | *Ace-1* | GGAATCCGTTTAGCAGAAGC | AAAAGCCCCGTCAATAATCG |
|  | *Chit* | CCGCGTCCCTATACCTAATC | TTCTGTGGCTTTTGTAACGC |
|  | *EF-1a* | GCAACTCCTTGACTGAAACG | AAATCGGTGGTATCGGAACG |
|  | *Pix* | ATGACTTTAGCAGCAACCAA | AGATGTAATGAAGCCAATGA |
|  | *Tw* | TATTACTCTTGGCCAACCCG | GAATAGTGGGCTTTTCGACG |

^a^ For full names, see Supplementary Table S5.

**Supplementary Table S5**. List of degenerate primers used for the cloning of selected reference and marker genes.

| Gene | Symbol | Forward primer | Reverse primer | Frag. size | Tm [°C] |
| --- | --- | --- | --- | --- | --- |
| *90-kDa heat shock protein* | *Hsp90* | F01-ATGATHGGNCAR TTYGGNGT | R01-GCRTGNACYTGNGGYTCRTC | 1644 | 50-60 |
|  |  |  | R02-TGRTCYTCCCARTCRTTNGT | 555 | 50-60 |
| *Acetylcholinesterase 1* | *Ace-1* | F01-ATHCCNTAYGCNCARAARCC | R01-GCRAARTTNGCCCARTAYCTCAT | 1334 | 55 |
| *chitinase h* | *Chit* | F01-TAYTTYGTNGARTGGGGNGT | R01-TTRTCNGCRTCDATYTCCCA | 1148 | 50-60 |
| *Elongation factor 1 alpha* | *EF-1a* | F01-AARGARGCNCARGARATGGG | R01-GCNACNGTYTGNCGCATRTC | 1172 | 58-60 |
| *pixie ATP-binding cassette sub-family E member 1* | *Pix* | F01-CARTAYGTNGAYCARATHCC | R01-01GCCATDATRAARTCRTGYTC | 1067 | 55-58 |
|  |  | F02-ATHTTYATGTTYGAYGARCC |  | 869 | 50-60 |
| *twitchin* | *Tw* | F01-CARGARGAYGAYGGNAAYAA | R01-GCYTTNACYTTRCANCCCAT | 1677 | 50-60 |
|  |  | F02-GGNGAYAARCCNAARATHCC |  | 732 | 50-60 |

Frag. size, expected fragment size; Tm, melting temperature.

**Supplementary Table S6**. Karyotype numbers in Gelechioidea.

| Family | Subfamily | Tribe | Species | M  n | F  n | M 2n | F  2n | n^a^ | Large chromosome pair present | Reference(s) |
| --- | --- | --- | --- | --- | --- | --- | --- | --- | --- | --- |
| Gelechiidae | Anacampsinae | Anacampsini | *Anacampsis disquei* | 29 |  |  |  |  | Yes | Lukhtanov and Kuznetsova 1989 |
|  |  |  | *Anacampsis innocuella* | 29 |  |  |  |  | Yes | Ennis 1976 |
|  |  |  | *Anacampsis populella* |  |  |  |  | 29 | Yes | Beliajeff 1930 (as *Tachyptilia populella*) |
|  | Apatetrinae | Pexicopiini | *Pectinophora gossypiella* | 30 | 30 |  |  | 30 | Yes | Bartlett and Lewis 1973  Bartlett and Del Fosse 1991 |
|  |  |  | *Pectinophora scutigera* |  |  | 60 |  |  | Not described | LaChance and Ruud 1979 |
|  |  |  | *Sitotroga cerealella* | 30 | 30 | 60 | 60 |  | Yes^b^ / No^c^ | Lukhtanov and Kuznetsova 1989  This work |
|  | Gelechiinae | Gnorimoschemini | *Tuta absoluta* | 29 | 29 | 58 | 58 |  | Yes | Carabajal Paladino *et al.* 2016  T his work |
|  |  |  | *Phthorimaea operculella* | 29 | 29 | 58 | 58 |  | Yes | Bedo 1984  Makee and Tafesh 2006 |
|  |  | Litini | *Coleotechnites piceaella* | 30 |  |  |  |  | Yes | Ennis 1976 (as *Pulicalvaria piceaella*) |
|  |  |  | *Coleotechnites resinosea* | 30 |  |  |  |  | Yes | Ennis 1976 (as *Eucordylea resinosae*) |
|  |  |  | *Coleotechnites* sp. | 29 |  |  |  |  | Yes | Ennis 1976 (as *Eucordylea* sp) |
|  |  |  | *Exoteleia dodecella* | 12 |  | 24 | 23 |  | Not described | Ennis 1976 |
|  |  |  | *Exoteleia nepheos* | 12 |  | 24 | 24 |  | Not described | Ennis 1976 |
|  |  |  | *Exoteleia pinifoliella* | 11 |  | 22 | 21 |  | Yes | Ennis 1976 |
| Coleophoridae | - | - | *Coleophora binderella* | 29 |  |  |  | 29 | Yes | Puplesiene 1993  Lukhtanov and Puplesiene 1999 |
|  |  |  | *Coleophora follicularis* |  |  |  |  | 40 | Yes | Lukhtanov and Puplesene 1996  Lukhtanov and Puplesiene 1999 |
|  |  |  | *Coleophora glitzella* | 29 |  |  |  | 29 | Yes | Puplesiene 1994  Lukhtanov and Puplesiene 1999 |
|  |  |  | *Coleophora laricella* | 29 | 29 | 58 | 58 |  | Yes | This work |
|  |  |  | *Coleophora lutipennella* |  |  |  |  | 57 | Yes | Puplesiene 1993  Lukhtanov and Puplesiene 1999 |
|  |  |  | *Coleophora ornatipennella* |  | 29 |  |  |  | Not described | Sruoga and Puplesiene 1998 |
|  |  |  | *Coleophora serratella* | 28 |  |  |  | 28 | Yes | Puplesiene and Puplesis 1992  Puplesiene 1993  Lukhtanov and Puplesiene 1999 |
|  |  |  | *Coleophora sibiricella* | 29 |  |  |  | 29 | Yes | Puplesiene 1994  Lukhtanov and Puplesiene 1999 |
|  |  |  | *Coleophora spinella* | 57 |  |  |  | 57 | Yes | Lukhtanov and Puplesiene 1996  Lukhtanov and Puplesiene 1999 |
|  |  |  | *Coleophora tadzhikiella* | 52 |  |  |  | 52 | Yes (two pairs) | Puplesiene 1993  Lukhtanov and Puplesiene 1999 |
|  |  |  | *Coleophora vitisella* | 29 |  |  |  | 29 | Yes | Puplesiene 1994  Lukhtanov and Puplesiene 1999 |
| Elachistidae | - | - | *Elachista adscitella* |  |  |  |  | 29 | Yes | Puplesiene 1993 (as *Elachista revinctella*) |
|  |  |  | *Elachista bifasciella* |  |  |  |  | 31 | Yes | De Prins and Saitoh 2003 |
|  |  |  | *Elachista maculicerusella* |  |  |  |  | 29 | Not described | Puplesiene 1994 (as *Elachista cerusella*) |
|  |  |  | *Perittia tectusella* |  |  |  |  | 30 | Yes | Puplesiene 1994 (as *Perittia weberella*)  Sruoga and Puplesiene 1998 |
|  |  |  | *Tonica niviferana* |  |  |  |  | 30 | Not described | Kaur 1988 |
| Xylorictidae | - | - | *Metathrinca tsugensis* |  |  |  |  | 30 | Yes | Kawazoé 1987 |
|  |  |  | *Opisina arenosella* | 30 | 30 | 60 | 60 |  | No | This work |
| Oecophoridae | - | - | *Hofmannophila pseudospretella* | 28 | 28 | 56 | 56 |  | Yes | This work |
| Depressariidae | - | - | *Agonopterix nervosa* | 30 |  |  |  |  | Not described | Regnart 1933 (as *Depressaria nervosa*) |
|  |  |  | *Depressaria daucella* | 30 | 30 | 60 | 60 |  | No | This work |
|  |  |  | *Nites grotella* | 29 |  |  |  |  | Yes | Ennis 1976 (as *Depressaria grotella*) |
|  |  |  | *Psilocorsis quercicella* |  |  | 54 |  |  | Yes | Ennis 1976 |

^a^ Sex not specified.

^b^ Lukhtanov and Kuznetsova 1989

^c^ This work

**References**

Bartlett AC, Del Fosse FE. 1991. The pachytene karyotype of the pink bollworm (Lepidoptera: Gelechiidae). Southwest Entomol*.* 16:223–235.

Bartlett AC, Lewis LJ. 1973. Pink bollworm: Chromosomal damage and reproduction after gamma irradiation of larvae. J Econ Entomol. 66:731–734.

Bedo DG. 1984. Karyotypic and chromosome-banding studies of the potato tuber moth, *Phthorimaea operculella* (Zeller) (Lepidoptera, Gelechiidae). Can J Genet Cytol*.* 26:141–145.

Beliajeff NK. 1930. Die Chromosomenkomplexe und ihre Beziehung zur Phylogenie bei den Lepidopteren. Z Indukt Abstamm Vererbungsl*.* 54:369–399.

Carabajal Paladino LZ, et al. 2016. The effect of X-rays on cytological traits of *Tuta absoluta* (Lepidoptera: Gelechiidae). Fla Entomol*.* 99:43–53.

De Prins J, Saitoh K. 2003. Karyology and sex determination. In: Kristensen NP, editor. Handbook of Zoology. Lepidoptera, moths and butterflies. Berlin: de Gruyter. p. 449–468.

Ennis TJ. 1976. Sex chromatin and chromosome numbers in Lepidoptera. Can J Genet Cytol. 18:119–130.

Hebert PDN, Penton EH, Burns JM, Janzen DH, Hallwachs W. 2004. Ten species in one: DNA barcoding reveals cryptic species in the neotropical skipper butterfly *Astraptes fulgerator.* Proc Natl Acad Sci U S A*.* 1101:4812–14817.

Kaur T. 1988. Chromosome numbers of thirty-one species of Indian Lepidoptera. Genetica*.* 76:191–193.

Kawazoé A. 1987. The chromosome in the primitive or microlepidopterous moth-groups. I. Proc Jpn Acad. 63:25–28.

LaChance LE, Ruud RL. 1979. Interstrain and interspecific crosses between *Pectinophora gossypiela* and *P. scutigera.* J Econ Entomol*.* 72:618–620.

Lukhtanov VA, Kuznetsova VG. 1989. Karyotype structure in higher Lepidoptera (Papilionomorpha). Entomol Rev*.* 68:12–31.

Lukhtanov VA, Puplesiene J. 1996. Karyotypical peculiarities and main features of karyotype evolution in lepidopterans of the nepticuloid, tischerioid, gelechioid and tineoid complexes (Lepidoptera: Nepticuloidea, Tischerioidea, Gelechioidea s.l., Psychoidea-Gracillarioidea). Entomol Obozr*.* 75:310–323.

Lukhtanov VA, Puplesiene J. 1999. Polyploidy in bisexual Lepidoptera species (Insecta: Lepidoptera): Old hypotheses and new data. Bonn Zool Beitr*.* 48:313–328.

Makee H, Tafesh N. 2006. Sex chromatin body as a marker of radiation-induced sex chromosome aberrations in the potato tuber moth, *Phthorimaea operculella* (Lepidoptera: Gelechiidae). J Pest Sci*.* 79:75–82.

Puplesiene J. 1993. A provisional study of karyotypes of mining Lepidoptera. Acta Entomol Litu*.* 11:57–63.

Puplesiene J. 1994. Brief survey of karyotypes of mining moths (Lepidoptera). Entomol Rev*.* 73:17–25.

Puplesiene J, Puplesis R. 1992. Chromosomal study of *Coleophora serratella* (L.) (Lepidoptera: Coleophoridae). Acta Entomol Litu*.* 10:90–93.

Regnart HC. 1933. Additions to our knowledge of chromosome numbers in the Lepidoptera. Proc Univ Durham Phil Soc*.* 9:79–83.

Sruoga V, Puplesiene J. 1998. Contribution to the knowledge of central Asiatic microlepidoptera with description of new species. Acta Zool Litu*.* 8:38–48.

**
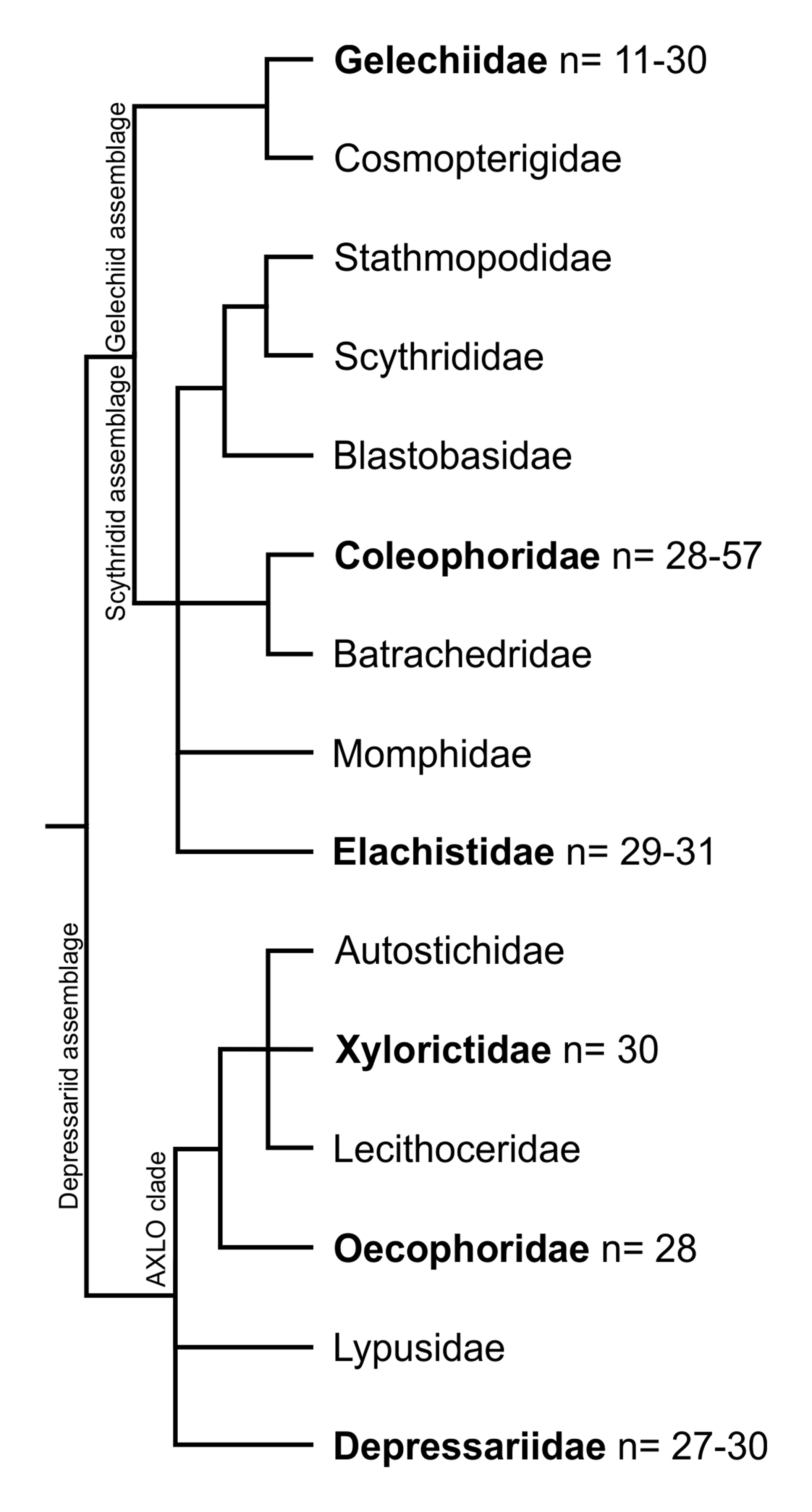
**

**Supplementary Fig. S1.** Phylogenetic tree of Gelechioidea *sensu* Sohn et al. (2016). Families in bold include species with known chromosome numbers.
